# A Case of Typhoid Fever, with Thoughts and Reflections on the Leading Features of the Pathology and Treatment of Low Continued Fevers

**Published:** 1871-09

**Authors:** Ch. Raushenberg

**Affiliations:** Atlanta, Georgia


ATLANTA

### MEDICAL AND SURGICAL JOURNAL.

NEW SERIES.

VOL. IX[illegible] ATLANTA, GEORGIA, SEPTEMBER, 1871. NO. 6.

### A CASE OF TYPHOID FEVER,

### WITH THOUGHTS AND REFLECTIONS ON THE LEADING FEATURES OF THE PATHOLOGY AND TREATMENT OF CONTINUED FEVERS.

By Ch. Raushenberg, M.D., Atlanta, Georgia.

I relate this (within itself not uninteresting) case as the most marked one amongst a number of others, where similar treatment was adopted and similar results obtained, and make it the subject of some remarks, because it first urged upon my mind the necessity of inquiring seriously into the propriety of using mercurials in these low types of fever, and formed the basis of a train of reflections in relation to this question and to the pathology and treatment of these diseases.

The use of mercurials in typhus and typhoid fevers—and I speak principally of their use in small doses—is not new. John Reid, Serres and others have used them with remarkable success, and the older systematic writers * recommend their use in these diseases. The prevailing opinion of the present day, however, is, that mercurials, as defibrinizers of

* Hamilton, DeLarogue, Wedemeyer, etc.

the blood, are injurious and inadmissible remedies in diseases characterized by a deficiency of fibrin in the blood; and their use is, therefore, *a priori*, condemned in low continued fevers by a large number of practitioners.

It has been my lot to see a number of cases where the results of this treatment encouraged the opinion that this is not absolutely correct; and I have ever since been endeavoring to harmonize these apparent contradictions between the teachings of theory and those of experience, and to arrive at a rational comprehension of the physiological reasons why mercurials have so often proved useful in the treatment of these diseases. In this effort, I have arrived at some conclusions, in relation to the general pathology of continued fevers, not generally entertained. Whether they be correct or erroneous, as the result of an honest search after truth,—be it even a weak one,—I expect for them charitable criticism.

I was called to see Dougherty H——, of Whitfield county, æt. fifteen. He had been treated previously, for five or six weeks, by Dr. Alston, of Red Clay, and Drs. Harrison, senior and junior, of Loudon, Tenn. At my request, Dr. Alston—who had abandoned the case as hopeless the day before—was called to give its history. He stated that enteric fever, with its usual symptoms, and with considerable tympanitis and diarrhœa, had existed for some length of time, but that the latter symptoms had finally yielded to liberal doses of opium and plumbum aceticum. The increasing prostration of the patient, in spite of stimulants and sustaining treatment, caused him to abandon the case as utterly hopeless.

The symptoms presenting themselves at my first visit were as follows: Coma nearly complete, low-muttering delirium, subsultus tendinum, features sallow and shrunken—at times, expressive of severe distress, great emaciation, skin cool, pulse very small, feeble, one hundred and twenty per minute, mouth dry, tongue narrow and pointed, covered with dark sordes in the middle, and with a narrow and thinner coat of evidently bilious origin around its edges, gums and teeth covered with dark sordes, abdomen sunk, presenting a concave surface, doughy on pressure—the convolutions of the small intestines well-definable as hard, unelastic tubes, with hard fæcal contents in the lower umbilical region—bowels have been costive for a number of days, patient turns quite restless occasionally, and then gives evidence of a desire to evacuate his bowels by distortion of the countenance and efforts to strain downwardly, right hypochondrium tender to pressure; on close examination, the conjunctiva shows a yellowish turbidity.

These symptoms indicating an almost complete inactivity of the secretory apparatus of the intestinal tube from the mouth downwards, an engorged and torpid state of the liver, and an accumulation of fæcal substances requiring evacuation, I thought it urgently necessary to meet these indications promptly, as the nerve-centers, already impaired by the original disease, must remain seriously depressed under this complicated functional derangement.

I proposed to administer a large dose of calomel, gr. x. to xv., to secure absorption of a sufficient quantity, as the most prompt, the least irritating, and, even in this case, the safest remedy to remove constipation and biliary engorgement; and afterwards to urge the desired restoration of the intestinal secretions by cautious administration of small doses of calomel, with opium and ipecacuanha, to stimulate the action of the heart, equalize the circulation, and restore the function of the skin.

Dr. Alston considered this treatment injurious, and withheld his consent. Dr. Harrison, junior, of Loudon, a relative of the family, who arrived that day, according to previous appointment, coincided with him; and castor oil and pulvis rhei were resorted to, to evacuate the bowels. These remedies were given in the evening and forepart of the night. The case remained unchanged; and in the forenoon of next day, at the request of the parents, I consented to take charge of the case, and treat it according to my views, with indeed but very little hope of success, but a conviction that I was doing justice to immediate indications in carrying out the above-mentioned propositions of treatment, which, as I reasoned, would relieve the small intestines of their long-retained and vitiated contents, probably improve the action of the liver, and, if continued in small doses, initiate secretory action throughout the alimentary canal—advantages which, if obtained, would certainly in this case, by their rectifying influences on the blood and nerve-centers, outweigh any general objections which might be raised against the use of calomel in an adynamic case on account of its impoverishing effect on the blood.

A large purgative dose of calomel was given in the forenoon, and followed by oleum ricini. Mild enemata were administered during the evening and the forepart of the night, and towards midnight a copious evacuation of hard, black lumps, with some thick, tenacious tar-like substance, was obtained, and soon followed by two others of a similar but less tenacious character.

After this the patient became quiet, the attacks of straining and restlessness ceased, the pulse decreased somewhat in frequency, and the coma and delirium diminished perceptibly. Brandy and strong beef essence were given at regular intervals, and in liberal doses. Next day, hydrargyr. mur. mit., gr. $\frac{1}{8}$, pul. ipecac., gr. $\frac{1}{3}$, pulv. opii, gr. 1-6, was given every three hours, and continued more or less regularly for nearly one week.

The coma and delirium diminished perceptibly under this treatment; the pulse increased in size and lessened in frequency; the skin became comfortably warm and moist, and the mouth and tongue moistened gradually; the sordes became loose and the tongue cleaned off; and with no other treatment but a liberal allowance of beef essence, good brandy and wine—and later, moderate doses of quinine and tepid baths—the patient was able to sit in a chair three weeks after my first visit, and soon afterwards regained his full health, and became heartier and fleshier than he had ever been before this attack of disease.

Did calomel, as it was used in this case, contribute to the restoration of the patient, or did he get well in spite of it? And is this remedy, as an alterative, admissible at all in low continued fevers? or does the pathology of these diseases forbid its use?

It is not my intention to investigate the effect of large doses of calomel, and to determine whether calomel really acts as a cholagogue proper, by a direct effect on the function of the liver, or not. No practitioner, however, of the smallest experience, can deny that a large dose of it almost invariably produces large bilious evacuations—whether in consequence of increased secretion of bile or in consequence of emptying the duodenum and upper intestines of the already-secreted bile before it can be absorbed again *—and that almost invariably a marked sedative effect is produced by this action. The accumulation of hard, bilious fæces, and the torpor of the liver—no matter what the true pathological condition of this organ may have been—was removed, and a marked improvement in the condition of the patient was perceptible in a remarkably short time after the calomel had exercised its purgative effect. It acted as a sedative; the delirium, restlessness, and frequency of the pulse abated, and the first step towards an improvement was made by the use of this, as it seemed, inadmissible and dangerous remedy.

The use of calomel, opium and ipecac, in very small doses, was next resorted to, with an expectation that the secretions of the mucous membrane of the alimentary canal, the liver and the skin, would thereby be restored; and their use was continued until a mild but perceptible impression to that effect was established. The exhaustion of the patient, the low degree of nerve-power, and the defibrinized condition of the blood, were, under the pressure of symptomatic indications for the use of mercury, left out of consideration, as contra-indications to its use, but were otherwise met by the simultaneous administration of brandy, beef-tea, quinine, etc.

The result of this practice was decidedly favorable, demonstrating that the vague idea, that a patient is too weak to bear a certain remedy or treatment, has certainly done harm occasionally, and has sometimes prevented the use of the most important remedies, by misapplying a general truth or principle of medicine to morbid conditions, without properly analyzing and interpreting their nature and origin.

The constitutional use of calomel in this case, notwithstanding the debility of the patient and the typhoid nature of

* See experiments of Bidder and Smith.

the case, by restoring the different secretions, was manifestly instrumental in the restoration of the patient's health.

Whether typhus and typhoid fever be identical, different species of one and the same disease, or generically different diseases, and whatever their exciting causes may be, the nature of their symptomatic development leaves no doubt in my mind that the effect of the poison which produces them is primarily exercised upon the blood, and that the growth and progressive development of this fluid within itself undergo the initial lesion in these diseases, and that, in consequence of this change in the blood, the whole process of vegetative (constructive) life, presided over by the sympathetic nerve, becomes irregular and morbidly deficient—absorption, nutrition, exchange of substance in the tissues, secretion and excretion deteriorate and become abnormal, and, with the increasing accumulation of effete material, the vitiation of the blood increases, the nerve-centers become deeply involved, and the disease secondarily becomes one of an apparently nervous character. *Thus, defective blood-formation and blood-development, with increased calorification and morbid oxygenation and combustion of tissue, form the prominent pathological features of low continued fevers.*

Hence, we find the quantity of fibrin and of red corpuscles in the blood of typhus and typhoid fever considerably decreased, that of the white corpuscles increased, the blood itself unusually dark and liquid, imperfectly oxygenized, while the urine of the patient is of a higher specific gravity, and contains more urea and uric acid, the products of nitrogenous combustion, than in health. (*a*)

A definite proportion of fibrin seems to be required to constitute healthy blood of normal consistency and constructive tendency. In sthenic inflammations and inflammatory fevers, the blood contains more, in asthenic fevers less fibrin than in health. If fibrin, then, as Virchow contends, is a product of lymphatic absorption, not increased in quantity in the blood by the inflammation of organs comparatively destitute of lymphatics—for instance, inflammation of the brain—and increased in quantity by bleeding and starvation, etc., is it not perfectly logical and correct to suppose that the formation of fibrin, and the introduction of the same into the blood, is directly proportionate to the degree of vitality with which the vegetative processes, the exchange of substance in the tissues, the absorption of the capillaries and lymphatics, are carried on?

In sthenic fevers, then, these processes must, generally speaking, be increased; in asthenic fevers, decreased.

What is the constitutional effect of mercurials on the system? Do they increase or decrease these processes of vegetative life? Do they increase or decrease the quantity of fibrin in the blood?

If mercurials are introduced in small doses into the healthy human system, where assimilation, absorption, exchange of substance, secretion and excretion are normally performed, all the secretions and excretions are abnormally increased in quantity first; and if their use is long enough continued, they are altered in quality—emaciation, so-called defibrinization of the blood takes place, and death results finally. If, however, these processes of absorption, secretion and excretion are deficient or abnormally decreased—as they are in low continued fevers—would the fact, that mercurials increase these functions, not justify the expectation, that the cautious use of mercurials in these fevers might be made instrumental in elevating these actions to the normal standard?

Why should the effect of mercurials not depend fully as much upon the physiological condition of the organs or tissues which they influence, in the performance of their function, as that of alcohol, opium, strychnine, ammonium muriaticum, etc.? And can we not, if we carry the effect of these remedies beyond the restoration of normal physiological action, produce the same or similar morbid conditions as those which we cure by their use, when this action is below the normal standard?

Do we not cure and also produce delirium by alcohol? Do we not allay spasms by opium, or produce them, if we use it improperly? Can we not cure paralysis by strychnine, or produce it, if we continue its use too long? Can we not occasion, as well as arrest, uterine hemorrhage by ergot? Can we not cure, as well as create, irritation and inflammation of the mucous membranes of the alimentary canal or respiratory tubes by muriate of ammonia? Why, then, is it improper to suppose that mercury, where the process of absorption and blood-formation is below the normal degree of activity, can can be made to increase this process, and increase the quantity of fibrin in the blood, and act as a fibrinizer, while, as soon as this effect is carried beyond the normal degree, hyperabsorption and hypersecretion induce a process of defibrinization, and it becomes a defibrinizer?

I have, therefore, arrived at the conclusion, that mercurials, while they can certainly not be recommended as febrinizers of the blood in continued fevers, should be used without hesitation whenever the condition of the liver or the intestinal mucous membrane offer plain and unmistakable indications for their use; and that the common idea, that as defibrinizers they are inadmissible in the treatment of low continued fevers, is, pathologically, not strictly correct.

Besides these evidences of diminished activity of the organs, necessary for the formation and maintenance of normal blood—to wit, absorption and secretion—we have to deal with another morbid process in continued fevers, which manifests itself in the increased quantity of urea and uric acid in the urine of these diseases.

The oxygenation of the blood continues with the process of respiration, but the nutrition of the tissues becomes excessively deficient in consequence of interrupted chylification. The oxygen of the capillaries enters into an abnormal process of combustion with the nitrogenous material of the tissues, and probably the muscular tissues principally, the products of which are necessarily eliminated by the kidneys in the form of urea and uric acid. (*b*) This process gives rise to an increase of the temperature of the body, and the greater the elevation of this temperature above the normal degree, the greater the combustion of tissue material—the greater the loss of force of the system. Thus, a patient lying in bed, but with a temperature of body five degrees higher than in health, is subjected, in one day, to a greater expenditure of force than a hard-working agricultural laborer in the field during the same length of time. (Houghton on Food and Force, "London Lancet.")

One of the greatest objects of the practitioner, in the treatment of typhoid and typhus fever, must, therefore, consist in lowering this process of abnormal combustion, and this object can be obtained—

1. By diminishing the temperature of the body, by frequently sponging the surface with cold water, wrapping the patient in wet sheets, or immersing him in a cold bath of seventy-seven degrees Farenheit once a day, if the bodily temperature in the axilla, or better, the anus, reaches one hundred and five degrees Farenheit. Traube, Wunderlich and Brande, of Germany, have highly recommended this so called antipyretic treatment, and claim to have attained by it a considerable decrease of the mortality of typhoid fever. They generally give a glass of good wine after the bath, and from fifteen to twenty grains of quinine, dissolved in water and muriatic acid, at bed-time every other night; and claim for this remedy a so-called antipyretic effect,—or, in other words, the effect that it restricts the nitrogenous decomposition of the tissues by the oxygen of the blood and the formation of urea in the urine.

2. By diminishing the frequency of the pulse, and with it the motion of the blood in the capillaries; hence the quantity of oxygen which comes in contact with the tissue-molecules. This may, in many instances, be accomplished by maintaining the pulse at eighty to eighty-five per minute, by the cautious use of tinctura veratri viridis. The depressing effect of this remedy, where the action of the heart is not intermittent, and the first sound not abnormally weak, need not be feared. I have used this remedy in well-marked cases of typhoid fever for days, and even weeks, in succession, generally combined with alcohol, and now and then with morphine or tinctura opii, not only without any ill, but with decidedly beneficial effect.

3. By furnishing nitrogenous substances, as well as hydrocarbons to the blood, to save tissue-material necessary for the integrity of the body.

This is certainly one of the most, if not the most, important object in the treatment of these diseases from the very commencement. We must generally depend upon absorption in the stomach for the introduction of these materials in the blood, as the mucous membrane of the small intestines is too deeply diseased to expect absorption by her lacteals; hence, they must be of a fluid character—alcohol diluted with water, extract of malt, and beef-essence,—the first two to supply hydrocarbons, the latter one to supply nitrogenous material. Frictions of the surface of the body with animal fat, so highly recommended by a few, come also under this head, as they furnish fat (hydrocarbon) to the blood by capillary absorption through the skin.

---

(*a*) The frequent manifestation of disease in Brunner's and Peyer's and the mesenteric glands, in typhoid fever, is certainly an additional evidence of the general functional derangement of this system of vessels in this disease.

If the lymphatics and lacteals, (the latter only when not engaged in the transportation of chyle), after absorbing lymph from the tissues, convert the albumen and the white corpuscles of this fluid, by bringing it in contact with the granular substance and the oxygen of the afferent vessels of their glands, relatively into fibrin and red corpuscles, a diseased condition of these vessels and glands and a diminished activity of their function would not only diminish the quantity of lymph absorbed, but also interfere with its partial transformation into fibrin and red corpuscles during its passage through these vessels.

(*b*) If, in severe cases of low continued fevers, all the actions of vegetative life are reduced to a minimum, while a deficient absorption, a morbid exchange of substance, an imperfect development of blood, and an increased but abnormal combustion and disintegration of tissue and elimination of its products principally as urea, constitute the essential factors of the morbid process of low continued fevers, this *morbid chemical process*, carried on in the laboratory of the body, might, at the suggestion of my friend, Mr. Th. Schuman, of this city, be demonstrated to the mind by deducting from the chemical formula of protein that of urea, multiplied by two, as one equivalent, in its decomposition, would, as is often the case in chemical decompositions, probably form two equivalents of urea, to wit:

$$C_{36}\ H_{25}\ N_4\ O_{10}\ \text{(protein according to Mulder.)}$$

$$C_4\ H_9\ N_4\ O_4\ \text{(urea.)}$$

$$\overline{C_{32}\ H_{17}\ —\ O_4}$$

The formula resulting from this deduction seems to represent mentally, approachingly correct, the true state of affairs in the system, as it leaves, as the result of this decomposition of the protein molecules into urea, only a certain quantity of carbonic acid and hydrogen to be eliminated through the lungs, and still a remnant of oxygen in the tissues to increase morbid combustion—thus indicating that, even if no other pathological phenomena would end life, this internal process of disintegration and combustion would, after a certain time, necessarily put an end to the patient's life by syncope.

